# Reforms

**Published:** 1894-04

**Authors:** Sigel Roush


### Reforms.

BY SIGEL ROUSH, A.M., M.D., D.D.S.

Assistant Demonstrator, Dental Department of the Columbian University, Washington, D.C.

When a young man receives his degree he immediately sets about to work great reforms in his chosen field of labor. He feels he has no time to lose, but must act promptly and with decision. He discerns grave and menacing defects and appalling errors that threaten his profession with complete annihilation. He feels he is destined to perform a great work and is impatient for the fray. He usually inaugurates this reformatory measure by addressing to the leading organ of his profession an exhaustive and verbose treatise, in which he learnedly reprobates existing evils, freely gives much-needed advice, and urges with the ardor and ignorance of a parvenue his gigantic and idealizing schemes. The young divine fresh from the seminary suggests ways and means by which the world may be rapidly transformed into a veritable millennium; the beardless but confident young disciple of ÆSCULAPIUS would dazzle the world by the brilliancy of his reforms in the healing art; the young legal limb would put Blackstone to shame by his wonderful forensic achievements and his original interpretations and applications of law; and the full-fledged "knight of the masticatory organs" beholds, but for his timely advice, the noble art of dentistry sink into odium and degradation. Your writer belongs to the last-named profession. He has been dubbed "D.D.S.," and is, consequently, prepared to inform the hoary-headed members of his fraternity how to conduct themselves in a manner that will redound to the glory and honor of dentistry.

To begin at the beginning, dentists, like poets, are born not made. The boy who would rather spend his Sundays in his father's workshop, where he has some mechanical apparatus in course of construction, than go to Sunday-school will make a better dentist than the young prodigy who had read all of Dickens and could repeat half of Shakespeare before he had reached the age of twelve.

Most dentists, I believe, resent the term "mechanic" or "mechanical" when applied to their profession, and yet, in the opinion of your writer, it would be easier for the proverbial camel to pass through the eye of a needle than for a dentist to attain any considerable measure of success unless he possessed, in a marked degree, mechanical skill of the finest quality. In nine out of every ten cases that come to a dentist's office, mechanical skill alone is all that is necessary to proficient and professional work. Why should the charge then that we are mechanics so ruffle us when it is the highest recommendation we can possess to guarantee valuable services? Who would not prefer to be a first-class mechanic to a third-class member of a profession? 'Tis true, a knowledge of anatomy, pathology, hygiene and therapeutics in operative and surgical dentistry, and of the properties and uses of the material employed in prosthetic dentistry, is, of course, necessary; but with this knowledge alone the would-be dentist is a decided failure, and when I see an awkward, blundering student, without even a suggestion of mechanical skill or ingenuity in his composition, but who has chosen dentistry for his life work because he knows Dr. So-and-So makes from three to seven thousand dollars a year in dental practice, my heart goes out in sincerest pity for him. He will grow to hate his profession, and after having failed to subsist upon his income and lost years of his valuable time he may realize his mistake, and finally find work for which he possesses some natural ability. Without love for and interest in his chosen work, success in the highest and noblest sense can never be obtained.

America is the home of dentistry, and American dental colleges are the best in the world, but even here some reforms might be instituted with salutary effect on the profession. Many colleges have short and incomplete courses. The faculties not paid at all or receive meagre compensation for their services, and only accept their chairs for the honor (?) of sitting on the stage at commencements and designating their positions on their letter-heads and business-cards. They occupy their chairs from selfish and mercenary motives, and, consequently, get through their course of lectures with as much ease and as little inconvenience to themselves as possible. The demonstrators in charge of the operating room and laboratory are either men with no practice, on account of youth and inexperience or on account of age and last century ideas. The infirmaries are often poorly equipped, and about all the average student learns in such a school is to make a monstrosity in the way of a rubber plate, and put in a half creditable amalgam filling. When he comes out of college, therefore, he is totally ignorant of modern, progressive dentistry. These same professors under whose proficient (?) and conscientious (?) services these students are graduated now organize private post-graduate courses and solicit matriculants. The new graduate is not long in realizing that his college course has been a farce, and that he is totally unprepared to practice intelligently modern dentistry, and joins these private classes where he sometimes learns for a liberal fee what should have been taught him for his college tuition.

Infirmaries, again, are often made money-making institutions. "Free dentistry, except cost of material, under the direction of skillful operators" is advertised, and by using material furnished at a nominal price by houses anxious to introduce their goods, and by careless and rapid work, and by admitting indiscriminately all who apply, the daily net earnings of an ordinarily well-patronized infirmary are often more than those realized from an average office practice. The writer has known the children of wealthy parents to come to the infirmary to have their teeth filled. On inquiry in one of these cases, it was learned that the child, a girl of fifteen, had been instructed to go to the family dentist and have him do all the work that was necessary in order to put her teeth in first-class order. She was supplied with sufficient money to pay her bill. The girl, however, had an eye to business and went instead to the infirmary, where she had her work done, and after paying her bill she pocketed about ten dollars extra money, which she explained would take her to the matinee at least ten times with a little left for candy. In another case the writer saw a well-dressed lady drive up to the infirmary door, instruct her coachman to return in an hour, enter the college, and have her teeth fixed. Her husband owned valuable property and was well-to-do.

Admission to the infirmaries should be regulated, and no work should be done for any one who is able to pay regular prices. If blanks were placed in the hands of the various charitable organizations of our cities with instructions to fill in the names of any charity patients who were in need of dental services, and then only admit patients who held these cards, it is the opinion of your writer that the infirmaries would have sufficient material without robbing the reputable dentists of their rightful patients. Dental associations and other fake organizations have undoubtedly injured legitimate practice. Every city has its cheap advertising dentists. Plates are made for from three to eight dollars. As a result, plate-work especially has suffered a falling off. Many well-to-do and intelligent people will give themselves credit for five or ten dollars and take their chances in getting a satisfactory plate of these cheap dentists. Is there a remedy to this menace to legitimate prices? It is a difficult question with which to deal. The operators are, as a rule, graduates from reputable colleges, and are consequently legally entitled to practice. Perhaps regular dentists are more or less responsible for this state of affairs. In the first place, dental societies are not made sufficiently attractive to induce new practitioners to join. In some cases the fees are an object. The older dentists take little or no interest in the new members. Many established dentists are selfish and mercenary, and instead of welcoming young members into their ranks resort to every means to starve them out. If they have work to throw into the hands of other dentists they prefer to give it to an older member—one who is on a living basis and who is hopelessly established. Our professors *at commencement* welcome us to the profession in the most unselfish and fraternal words, and take every opportunity to keep us out of practice. Happily, there are exceptions to this rule.

Finally, the young dentist becomes soured and discouraged. He can't live on ethics, and having already learned that he will receive no help from the established dentists of his city he adopts advertising and cheap work, and so-called reputable dentists are ften responsible for the course he has chosen.

Again, we lack organization and harmony. Unless we stand as a body we will never be able to maintain scheduled prices. Our societies often don't include half the practicing dentists of the place. Lack of interest has made them unattractive. Let us build up and increase the membership of our organizations. Give the new members, a chance. Appoint them on commitees and let them read papers. Throw enough work into their hands to keep them on their feet, and your writer believes we will have fewer to desert, or rather we will have driven fewer members into the ranks of charlatans and quacks.

